# Release of Ca2+ and Mg2+ from yeast mitochondria is stimulated by increased ionic strength

**DOI:** 10.1186/1471-2091-7-4

**Published:** 2006-02-06

**Authors:** Patrick C Bradshaw, Douglas R Pfeiffer

**Affiliations:** 1 Institute on Aging, University of Wisconsin-Madison, Madison, WI, USA; 2 Department of Molecular and Cellular Biochemistry, Department of Internal Medicine, Institute for Mitochondrial Biology, The Ohio State University, Columbus, OH, USA

## Abstract

**Background:**

Divalent cations are required for many essential functions of mitochondrial metabolism. Yet the transporters that mediate the flux of these molecules into and out of the mitochondrion remain largely unknown. Previous studies in yeast have led to the molecular identification of a component of the major mitochondrial electrophoretic Mg^2+ ^uptake system in this organism as well as a functional mammalian homolog. Other yeast mitochondrial studies have led to the characterization of an equilibrative fatty acid-stimulated Ca^2+ ^transport activity. To gain a deeper understanding of the regulation of mitochondrial divalent cation levels we further characterized the efflux of Ca^2+ ^and Mg^2+ ^from yeast mitochondria.

**Results:**

When isolated mitochondria from the yeast *Saccharomyces cerevisiae *were suspended in a salt-based suspension medium, Ca^2+ ^and Mg^2+ ^were released from the matrix space. Release did not spontaneously occur in a non-ionic mannitol media. When energized mitochondria were suspended in a mannitol medium in the presence of Ca^2+ ^they were able to accumulate Ca^2+ ^by the addition of the electrogenic Ca^2+ ^ionophore ETH-129. However, in a KCl or choline Cl medium under the same conditions, they were unable to retain the Ca^2+ ^that was taken up due to the activation of the Ca^2+ ^efflux pathway, although a substantial membrane potential driving Ca^2+ ^uptake was maintained. This Ca^2+ ^efflux was independent of fatty acids, which have previously been shown to activate Ca^2+ ^transport. Endogenous mitochondrial Mg^2+ ^was also released when mitochondria were suspended in an ionic medium, but was retained in mitochondria upon fatty acid addition. When suspended in a mannitol medium, metal chelators released mitochondrial Mg^2+^, supporting the existence of an external divalent cation-binding site regulating release. Matrix space Mg^2+ ^was also slowly released from mitochondria by the addition of Ca^2+^, respiratory substrates, increasing pH, or the nucleotides ATP, ADP, GTP, and ATP-gamma-S.

**Conclusion:**

In isolated yeast mitochondria Ca^2+ ^and Mg^2+ ^release was activated by increased ionic strength. Free nucleotides, metal ion chelators, and increased pH also stimulated release. In yeast cells this release is likely an important mechanism in the regulation of mitochondrial matrix space divalent cation concentrations.

## Background

Mg^2+ ^is the most abundant divalent cation in cells and is important for many metabolic processes. Free Mg^2+ ^in cells is quite low (0.3 – 3%) as compared to the total amount of Mg^2+ ^due to buffering by polyphosphates, nucleic acids, and free nucleotides [1,2]. In mammalian cells the free concentration is usually maintained at a concentration between 0.25 and 1 mM [2]. The Mg^2+ ^level in the cell varies during changing physiological conditions. The free Mg^2+ ^level increases slightly as ATP levels decrease. The total cellular Mg^2+ ^level changes upon the addition of several hormones to cells [3]. Recently the TRPM family of ion channels has been found to play an important role in cellular Mg^2+ ^homeostasis in several tissues [4]. This suggests that regulated pathways for Mg^2+ ^transport exist to maintain proper intracellular levels.

### Mammalian mitochondrial Mg^2+ ^transport

The pathways involved in mammalian mitochondrial Mg^2+ ^flux are largely unknown. The human homolog of a component of the main electrophoretic mitochondrial Mg^2+ ^transporter in yeast has been cloned [5]. The protein can functionally substitute for its yeast homolog. These double membrane-spanning proteins likely homo-oligomerize to form ion-transporting complexes. However definitive evidence that this protein functions in mammalian mitochondrial ion transport has yet to be obtained.

Heart mitochondria take up and release Mg^2+ ^in a respiration-dependent manner [6-8]. Uncouplers of respiration that dissipate the membrane potential release mitochondrial Mg^2+ ^into the cytoplasm [9]. There is evidence that hormones alter mitochondrial Mg^2+ ^levels [10]. Hormones may increase cyclic AMP (cAMP) levels to stimulate Mg^2+ ^transport [11]. Fatty acids have been shown to stimulate the efflux of mitochondrial Mg^2+ ^at alkaline pH [12]. Even though mitochondrial Mg^2+ ^has been well characterized at the isolated organelle level, the cellular signals regulating mitochondrial Mg^2+ ^transport have not been clearly elucidated.

### Mg^2+ ^transport in yeast

In yeast cells when medium Mg^2+ ^is plentiful (> 1 mM), yeast cells contain around 400 nmole Mg^2+^/mg protein [1]. As medium Mg^2+ ^decreased (0.02 mM), cellular Mg^2+ ^decreased to levels near 80 nmole/mg protein. In times of ample Mg^2+^, the Mg^2+ ^is taken up and stored in the vacuole. When these cells were then placed in a Mg^2+ ^free growth medium, they survived by utilizing their vacuole stores. But once the cellular Mg^2+ ^level became too low, the yeast cells died, in contrast to limitations in many other nutrients in which the cells survive in the G_0 _phase of the cell cycle [1].

Two genes comprising the yeast Mg^2+ ^uptake system in the plasma membrane have been identified [13]. Yeast plasma membrane Mg^2+ ^transport is inhibited by aluminum [13] and polyamines [14]. Since overexpression of either of the Mg^2+ ^transport proteins confers resistance to aluminum toxicity, the inhibition of Mg^2+ ^uptake may be the primary cause for aluminum toxicity in yeast [13]. Mg^2+ ^may also provide benefits to cells confronted with heavy metals. Increased cellular Mg^2+ ^levels protected cells from toxic concentrations of Mn^2+ ^by down-regulating Mn^2+ ^transport [15].

### Yeast mitochondrial Mg^2+^

Mg^2+ ^is essential for many mitochondrial reactions including those of DNA and RNA metabolism [16,17] as well as ATP synthesis [18]. Mg^2+ ^blocks the yeast mitochondrial ATP-induced unspecific channel (YMUC) [19]. Mg^2+ ^also inhibits mitochondrial anion uniport [20]. Therefore, the regulation of matrix space Mg^2+ ^could be important for the mitochondrial distribution of many other ions. In this report we discovered that Mg^2+ ^and Ca^2+ ^are released from yeast mitochondria under conditions of increased ionic strength. In yeast cells this release pathway is likely an important regulator of mitochondrial divalent cation levels.

## Results

### Ionic media prevent ETH-129-mediated accumulation of mitochondrial Ca^2+^

**Figure 1:**
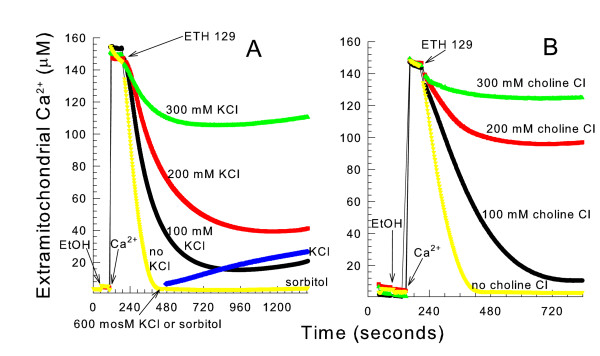
**ETH-129 mediated mitochondrial Ca^2+ ^uptake is greatly reduced in an ionic medium**. *panel A*, the medium contained a 0.6 osmolar (osM) mixture of 0.3 M KCl and 0.6 M mannitol, 10 mM HEPES (TEA^+^), pH 7.20, 10 mM KP_i_, 0.5 mg/ml BSA, 10 μM EGTA (K^+^), and 0.1 mM antipyrylazo III. Where indicated 1 mM ethanol, 150 μM CaCl_2_, 3.6 μM ETH-129, 0.3 M KCl, and 0.6 M sorbitol were added. *panel B*, the conditions were the same as *panel A*, except 0.3 M choline Cl was present instead of KCl.

In contrast to mammalian mitochondria, energized yeast mitochondria do not elecrophoretically take up Ca^2+ ^or undergo an increase in non-specific inner membrane permeability in the presence of Ca^2+ ^[21]. However energized yeast mitochondria open an unspecific channel (YMUC), which dissipates the membrane potential in the absence of the YMUC inhibitors phosphate or decavanadate. Yeast mitochondria can accumulate Ca^2+ ^by the addition of the electrogenic Ca^2+ ^ionophore ETH-129 to intact cells [22] or to energized mitochondria in a mannitol medium [21]. When suspended in a 0.3 M KCl medium, energized mitochondria were unable to accumulate Ca^2+ ^after ETH-129 addition as measured by mitochondrial-targeted aequorin [22] or the presence of the Ca^2+^-indicating dye antipyrylazo III (Figure 1A). Less Ca^2+ ^was accumulated as the KCl concentration increased. Increasing the KCl concentration to 0.3 M only allowed less than 30 percent of the medium Ca^2+ ^to be accumulated. It is important to note that ETH-129 has no transport affinity for K^+ ^and the presence of KCl does not affect the binding of ETH-129 to Ca^2+ ^[23]. After all of the medium Ca^2+ ^was taken up by mitochondria suspended in a mannitol medium, the addition of 0.3 M KCl caused the slow efflux of Ca^2+ ^from the matrix space. This efflux of Ca^2+ ^did not occur when an equal amount of sorbitol was added. Similarly to mitochondria suspended in KCl media, mitochondria suspended in increasing concentrations of choline Cl media osmotically balanced with mannitol were increasingly unable to accumulate high levels of Ca^2+ ^by the addition of ETH-129 (Figure 1B).

**Figure 2:**
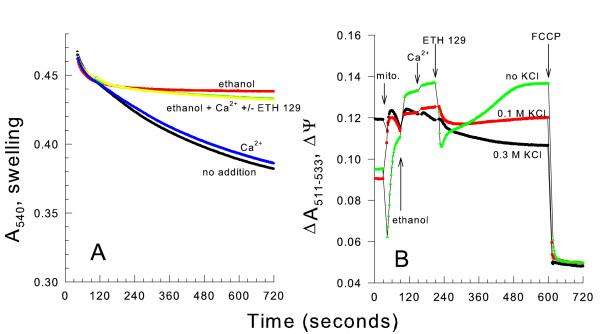
**Ca^2+ ^and ETH-129 don't cause swelling or a large ΔΨ drop when mitochondria are suspended in KCl**. *panel A*, the medium contained 0.3 M KCl, 10 mM HEPES, pH 7.20, 10 mM KP_i_, and 0.5 mg/ml BSA. 1 mM ethanol, 80 μM CaCl_2_, and 3.6 μM ETH-129 were present as shown. *panel B*, the medium contained a 0.6 osM mixture of 0.3 M KCl and 0.6 M mannitol, 10 mM HEPES (TEA^+^), pH 7.20, 10 mM KP_i_, 0.5 mg/ml BSA, and 12 μM safranine O. 1 mM ethanol, 80 μM CaCl_2_, 3.6 μM ETH-129, and 4 μM FCCP were added where indicated. 1 mM ethanol was chosen because this concentration prevents mitochondrial swelling in a 0.3 M KCl medium.

After suspending mitochondria in a 0.3 M KCl medium in the presence of Ca^2+^, ETH-129, and 1 mM ethanol (respiratory substrate), no mitochondrial swelling occurred (Figure 2A). Therefore no non-specific pore opened to allow Ca^2+ ^efflux or KCl influx. If a non-specific pore were to open, an influx of KCl into the matrix space would result in an increase in matrix space volume and a decrease in light scattering of the mitochondrial suspension. Further evidence for the lack of mitochondrial pore opening is that a substantial membrane potential (ΔΨ) was maintained in a KCl medium after ethanol, Ca^2+ ^and ETH-129 addition (Figure 2B). The ΔΨ was monitored by the absorbance of the dye safranine O [24].

In the KCl medium in the absence of Ca^2+ ^and ETH-129, the ΔΨ was roughly twenty percent smaller than that observed when the mitochondria were suspended in a mannitol medium. Immediately following Ca^2+ ^and ETH-129 addition the ΔΨ in the mannitol medium dropped to a lower value than that observed after the same treatment in the KCl medium. At the same time Ca^2+ ^was taken up more rapidly in the mannitol medium. Therefore a sufficient driving force for mitochondrial Ca^2+ ^uptake by ETH-129 was maintained in the KCl medium although the Ca^2+ ^was not retained in the organelle. The steady state ΔΨ attained in the 0.3 M KCl media after Ca^2+ ^and ETH-129 addition was approximately two-thirds of the value of that in the mannitol medium. These results are consistent with the activation of a Ca^2+^/H^+ ^exchange when mitochondria are suspended in the ionic media. This activity together with the electrogenic ETH-129-mediated Ca^2+ ^uptake causes proton re-entry into the matrix space to slightly decrease the ΔΨ. After the ΔΨ reached a steady state, it was completely dissipated by addition of the uncoupler FCCP (carbonyl cyanidep-trifluoromethoxyphenylhydrazone) to give the baseline level.

In a previous report a large decrease in the ΔΨ occured after ETH-129 addition to mitochondria suspended in a mannitol medium in the absence of BSA (bovine serum albumin) [25]. Together with ionophore-catalyzed Ca^2+ ^influx, activation of a fatty acid stimulated Ca^2+^/H^+ ^exchange led to the decreased ΔΨ. Because the ΔΨ was not dissipated to as great of an extent when mitochondria were suspended in a KCl medium, the Ca^2+ ^efflux pathway described here appears to be less active than the one activated by fatty acids [25].

### Increasing ionic strength induces release of Mg^2+ ^from mitochondria

**Figure 3:**
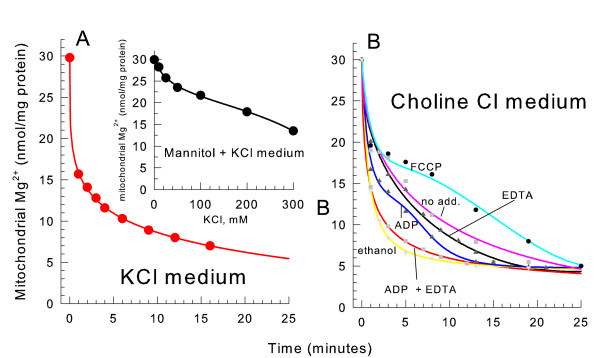
**An ionic medium induces a loss of mitochondrial Mg^2+^**. The medium contained 0.3 M KCl, 10 mM HEPES, pH 7.20, and 0.1 mM decavanadate (Na^+^). The mitochondria were spun down at the indicated time after suspension and analyzed for Mg^2+^. *inset*, the conditions were the same as *panel A *except the osmolarity of the medium was kept constant by replacing a portion of the KCl with mannitol. The mitochondria were spun down 4 minutes after suspension for analysis. *panel B*, the medium contained 0.3 M choline Cl, 10 mM HEPES (TEA^+^), pH 7.20, and 0.1 mM decavanadate (Na^+^). 4 μM FCCP, 2 mM ADP (Na^+^), 1 mM ethanol, and 2 mM EDTA (K^+^) were present where indicated.

Using atomic absorption spectrophotometry, we determined that isolated yeast mitochondria contain between 25 and 35 nmole Mg^2+^/mg protein. This is fairly consistent with a previously measured value of between 20 and 30 nmole Mg^2+^/mg protein [26]. When non-energized mitochondria were suspended in a 0.3 M KCl medium, endogenous Mg^2+ ^was lost from the matrix space in a time-dependent manner (Figure 3A). A slightly higher rate of Mg^2+ ^efflux also occurred in the presence of ethanol, ETH-129 and Ca^2+ ^under identical conditions to Figure 1A (data not shown). To study the effects of varied ionic strength on the Mg^2+ ^efflux rate, non-energized mitochondria were suspended in 0.6 osmolar (osM) media containing a varied KCl concentration osmotically balanced with mannitol (Figure 3A*inset*.) Increasing the KCl concentration increased the rate of Mg^2+ ^release from the matrix space. When no KCl was present virtually all of the Mg^2+ ^remained in the mitochondria.

Mg^2+ ^was also released from the matrix space when mitochondria were suspended in all other ionic media tested including K^+ ^TES, NaCl, and tetramethylammonium (TMA) Cl (data not shown). Endogenous Mg^2+ ^was released from the matrix space at slightly different rates in the different ionic media in increasing order TMA Cl < choline Cl < KCl < Tris Cl (compare K^+ ^in Figure 3A to choline in Figure 3B). These rates of Mg^2+ ^release corresponded to the rates of spontaneous swelling of yeast mitochondria in these salt media. However, Mg^2+ ^was released even faster than any of these spontaneous rates in a KCl medium in the presence of 1 mM ethanol (data not shown) where no swelling occurred (see Figure 2A). Mg^2+ ^was also released at a substantial rate in K^+ ^TES media where no swelling occurred. Therefore organelle lysis due to matrix space swelling was not responsible for the Mg^2+ ^release. The entry of ions into the matrix space may help displace bound Mg^2+ ^to stimulate the rate of efflux.

When non-energized yeast mitochondria were suspended in a choline Cl medium, the addition of either EDTA (ethylenediaminetetraacetic acid) or ADP slightly increased the Mg^2+ ^efflux rate (Figure 3B). The combined presence of EDTA and ADP increased the rate of Mg^2+ ^efflux more than either compound alone. The addition of ethanol as a respiratory substrate also increased the efflux of Mg^2+^. Since ethanol opens the yeast mitochondrial unselective channel (YMUC), decavanadate, a YMUC inhibitor [27,28], was present in the medium during all experiments. Since the efflux of Mg^2+ ^is fairly rapid even in the absence of ADP, EDTA, or ethanol, the addition of these compounds only slightly stimulated the rate of efflux.

### EDTA, respiration, ADP, and ATP stimulate loss of Mg^2+ ^from mitochondria

**Figure 4:**
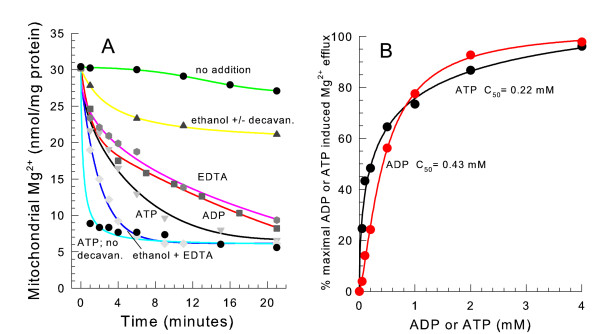
**ADP, ATP, ethanol, and EDTA stimulate mitochondrial Mg^2+ ^efflux in a mannitol medium**. *panel A*, the medium contained 0.6 M mannitol, 10 mM HEPES (TEA^+^), pH 7.20 and 0.1 mM decavanadate (Na^+^). Where indicated 1 mM ethanol, 2 mM ATP (Na^+^), 2 mM ADP (Na^+^), and 2 mM EDTA (K^+^) were present. *panel B*, the medium was the same as for *panel A*. The amount of mitochondrial Mg^2+ ^was measured 6 min. after suspension varying the ADP and ATP concentrations as indicated.

To verify the effects of the addition of EDTA, ethanol, and ADP observed in a choline Cl medium, similar experiments were performed suspending mitochondria in a mannitol medium where only a very small rate of spontaneous Mg^2+ ^release occured. As shown in Figure 4A, adding 2 mM ethanol only led to the release of 20–25 percent of the endogenous matrix space Mg^2+^. Adding 2 mM EDTA resulted in a quick initial and then subsequently slower release of Mg^2+^. The addition of EDTA and ethanol together led to a very quick release of almost all of the endogenous mitochondrial Mg^2+^. The addition of ATP caused a slightly greater rate of Mg^2+ ^release than ADP. The presence of carboxyatractyloside, an inhibitor of the adenine nucleotide translocator, did not alter the rate of Mg^2+ ^efflux when ATP or ADP was added. Therefore ADP and ATP likely bind to a site on the outer surface of the inner mitochondrial membrane to release Mg^2+^. ADP and ATP were half maximally effective at concentrations of 0.43 mM and 0.22 mM respectively (Figure 4B).

As described in Table 1, the nucleotides AMP and cAMP were not very effective at causing efflux of endogenous mitochondrial Mg^2+ ^in a mannitol medium. 0.1 mM EDTA was effective in releasing the majority of Mg^2+^, while 0.1 mM EGTA (ethylene glycol-bis-(beta-aminoethyl ether)-N, N, N', N'-tetracetic acid) was not effective. EDTA and EGTA are metal ion chelators. These results suggest that an inhibitory metal ion blocks the Mg^2+ ^release pathway at a site on the inner membrane facing the cytoplasm. EDTA likely binds the inhibitory metal ion to stimulate efflux. GTP and ATP-γS were nearly as effective as ATP in activating the Mg^2+ ^efflux pathway. The nucleotide analog 5'-fluorosufonylbenzoyladenosine (FSBA) partially blocked the ADP-induced Mg^2+ ^release suggesting that nucleotides do not activate release solely by chelating an inhibitory metal ion. The addition of 0.1 mM Ca^2+ ^also led to a slight increase in the Mg^2+ ^release rate. Unlike yeast plasma membrane Mg^2+ ^transporters [13], Al^3+ ^did not block mitochondrial Mg^2+ ^efflux (data not shown). La^3+ ^and ruthenium red, which block the Ca^2+ ^uniporter in mammalian mitochondria [29] were also unable to block Mg^2+ ^release in yeast mitochondria.

**Table 1:** Effectors of yeast mitochondrial Mg^2+ ^release. Yeast mitochondria were suspended in 0.6 M mannitol, 10 mM HEPES (TEA^+^), pH 7.20, and 0.1 mM decavanadate (Na^+^). Mitochondrial Mg^2+ ^was measured at 20 minutes. The percentage of Mg^2+ ^released was compared to that released in the presence 2 mM ATP (Na^+^).

**Effector**	% of 2 mM ATP induced Mg^2+ ^release
2 mM GTP	81.6
2 mM ATP gamma-S	89.3
2 mM ADP	88.7
2 mM AMP	19.1
0.2 mM cyclic AMP	5.2
2 mM FSBA	21.1
2 mM ADP + 2 mM FSBA	38.8
0.1 mM Ca^2+^	30.0
0.1 mM EGTA	4.8
0.1 mM EDTA	82.0

**Figure 5:**
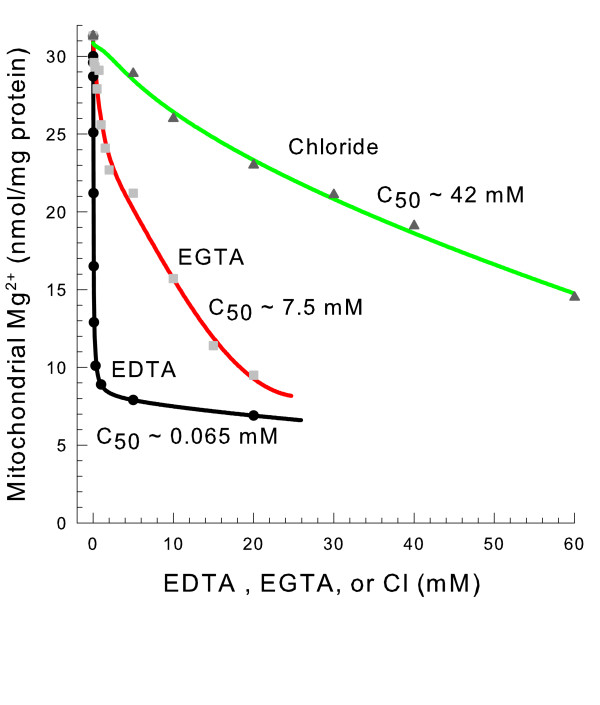
**EDTA stimulates mitochondrial Mg^2+ ^efflux much more potently than EGTA**. The medium contained 0.6 M mannitol, 10 mM HEPES (TEA^+^), pH 7.20, and 0.1 mM decavanadate (Na^+^). TEA EDTA, TEA EGTA, and TEA Cl were present as shown. Timepoints were taken at 20 minutes.

The concentrations of EDTA and EGTA added to the medium were varied to identify the amount of chelator needed to cause Mg^2+ ^release (Figure 5). 100 times more EGTA than EDTA was needed to cause an equal amount of Mg^2+ ^release. Both chelators stimulated efflux to a greater extent than an equal amount of chloride medium indicating that these compounds do not just release Mg^2+ ^by increasing the ionic strength of the medium.

### Fatty acids do not stimulate mitochondrial Mg^2+ ^efflux

**Figure 6:**
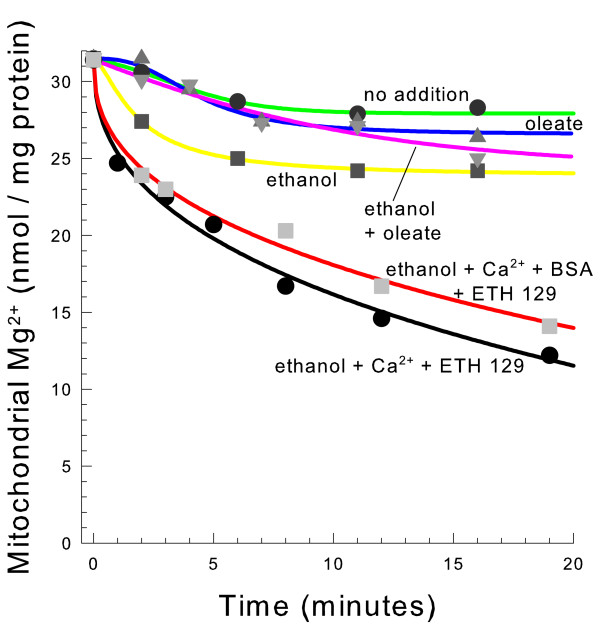
**Fatty acids do not stimulate Mg^2+ ^efflux from mitochondria**. The medium contained 0.6 M mannitol 10 mM HEPES (TEA^+^), pH 7.20, and 10 mM KP_i_. Where indicated 1 mM ethanol, 25 μM oleate (Na^+^), 80 μM CaCl_2_, 3.6 μM, ETH-129, and 1 mg/ml BSA were present. Mitochondria were spun down and analyzed for Mg^2+ ^by atomic absorption spectrophotometry.

Since fatty acids stimulate Ca^2+ ^transport from yeast mitochondria in a mannitol medium [25], we sought to determine if they also stimulate Mg^2+ ^release. We therefore performed experiments under the exact conditions which have previously been shown to stimulate Ca^2+ ^efflux from yeast mitochondria. As shown in Figure 6, free fatty acids or BSA, which binds free fatty acids, did not alter the rate of Mg^2+ ^efflux from the matrix space even in the presence of Ca^2+ ^and ETH-129 that were present in the past study. The presence of Ca^2+ ^slightly stimulated Mg^2+ ^efflux in a similar manner as is shown in Table 1. BSA or fatty acids also did not significantly alter the rate of mitochondrial Mg^2+ ^release when the mitochondria were suspended in a KCl medium (data not shown). This data suggest that the mitochondrial Mg^2+ ^efflux pathway studied in this report and the fatty acid-induced Ca^2+^/H^+ ^exchange are distinct activities.

### Increasing pH stimulates mitochondrial Mg^2+ ^efflux

**Figure 7:**
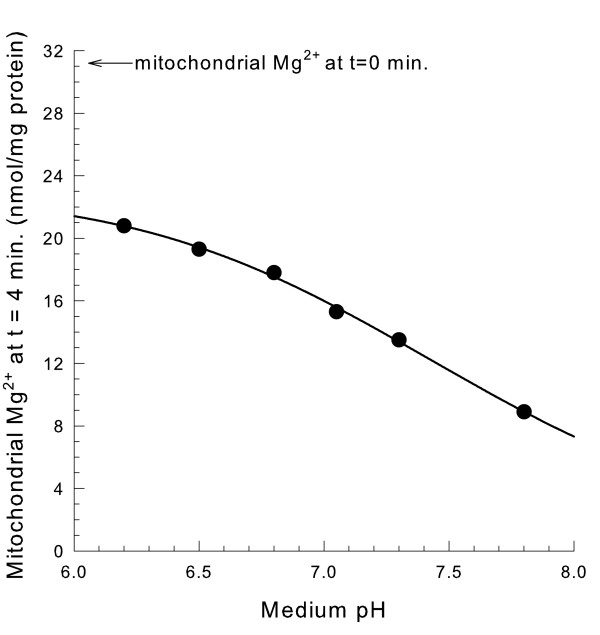
**Increasing pH increases mitochondrial Mg^2+ ^efflux**. The medium contained 0.3 M KCl, 10 mM HEPES for pH > 6.7 or 10 mM MES for pH < 6.7, the pH indicated, and 0.1 mM decavanadate (Na^+^). Mitochondria were spun down 4 min. after suspension and analyzed for Mg^2+^.

Increasing the pH increased the rate of Mg^2+ ^release from the mitochondrial matrix space in a KCl medium (Figure 7). The rate of release at pH 7.8 was nearly twice the rate at pH 6.5. This could be explained by the presence of a site for proton inhibition of Mg^2+ ^release. Protons may inhibit from the matrix space side of the inner membrane because pumping protons during respiration also stimulated Mg^2+ ^efflux.

### Mitochondrial divalent cation influx

**Figure 8:**
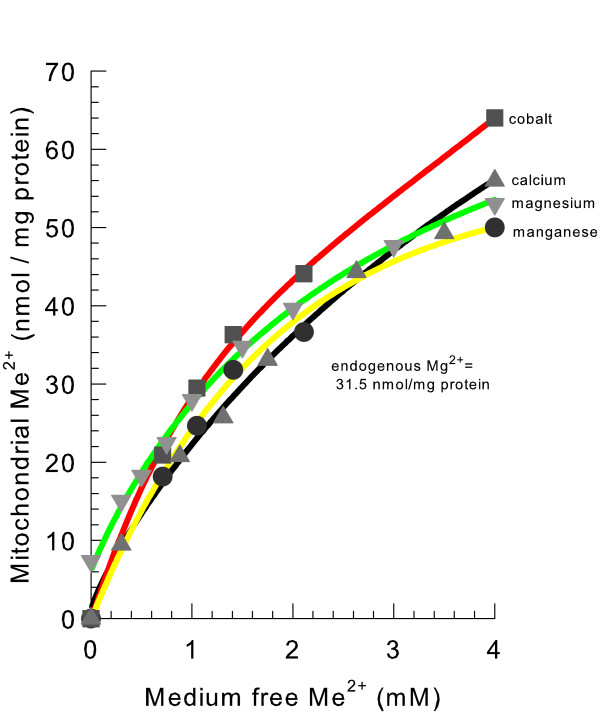
**Mg^2+^, Ca^2+^, Co^2+^, and Mn^2+ ^influx into yeast mitochondria**. The medium contained 0.3 M KCl, 10 mM HEPES, pH 7.2, and 0.1 mM decavanadate (Na^+^). Either 4 mM MgCl_2_, MnCl_2_, CoCl_2_, or CaCl_2 _were present as indicated. EGTA (K^+^) was present at different concentrations to yield the indicated free divalent cation concentrations as described in Methods. The mitochondria were incubated for 5 min. in the indicated free ion concentration, then spun down for 2 minutes in a microcentrifuge, washed once with a cold solution containing 0.6 M mannitol, 10 mM MES (TEA^+^), pH 6.0, and then spun down again. Ion concentrations were then determined as described in Methods.

To determine if divalent cation influx into non-energized yeast mitochondria could occur, the mitochondria were suspended in a KCl medium in the presence of increasing concentrations of the free divalent cations Ca^2+^, Co^2+^, Mg^2+^, or Mn^2+^. The total mitochondrial levels of the cations increased substantially (Figure 8). This increase is due to the large divalent cation buffering capacity present in the mitochondrial matrix space. This finding is consistent with previous results using mitochondrial-targeted aequorin indicating that free external and free mitochondrial Ca^2+ ^levels equilibrate [22]. Assuming that this nonspecific divalent cation influx activity also equilibrates free matrix space and external Mg^2+ ^concentrations, the free matrix space Mg^2+ ^concentration would be approximately 1.2 mM for our isolated mitochondria that contained 31.5 nmol Mg^2+^/mg protein. A free matrix space Mg^2+ ^level of 0.7 mM was measured in isolated yeast mitochondria using a fluorescent indicator [30].

## Discussion

The present study describes conditions in which isolated yeast mitochondria are able to release Mg^2+ ^and Ca^2+ ^from the matrix space. The efflux is most active when mitochondria are suspended in a high ionic strength medium. Respiration, ATP, ADP, EDTA, and Ca^2+ ^are also able to stimulate Mg^2+ ^efflux.

### The essential role of Mg^2+ ^in mitochondrial metabolism

Genetic studies in yeast have established that Mg^2+ ^is essential for mitochondrial RNA metabolism [17]. Mg^2+ ^also regulates the yeast mitochondrial ATP synthase [18]. The free concentration of Mg^2+ ^in beef heart mitochondria is estimated to be between 0.7 and 0.9 mM [10]. Within the physiological range, Mg^2+ ^can modulate the efficiency of the mammalian 2-oxoglutarate dehydrogenase and mitochondrial ATP synthase to affect the rate of oxidative phosphorylation [31]. Therefore the matrix space Mg^2+ ^level needs to be maintained within a specific range to maintain proper mitochondrial function. However in other species and tissues mitochondrial Mg^2+ ^has been shown to vary over a larger range from 0.2 to 1.5 mM [32,33].

### The role of EDTA and EGTA in stimulating Mg^2+ ^efflux

EDTA and EGTA have very different affinities for activating mitochondrial Mg^2+ ^release. The most likely candidate for a metal ion bound by EDTA and EGTA to activate Mg^2+ ^release is Mg^2+ ^itself because it has a greatly different affinity for each of these chelators. Many other divalent cations such as Ca^2+ ^do not satisfy this criterion. The ability of EDTA to activate mitochondrial Mg^2+ ^efflux from the cytoplasmic side of the inner membrane suggests that when the cytoplasmic concentration of the inhibiting ion is low, mitochondrial Mg^2+ ^equilibrates with the cytoplasmic concentration. However, the significance of mitochondrial Mg^2+ ^efflux functioning to supply Mg^2+ ^to the cytoplasm is questionable, since the vacuole is the major storage organelle for Mg^2+ ^in yeast cells [1].

The presence of a cytoplasmically facing Mg^2+ ^inhibiting the mitochondrial efflux of divalent cations does not appear to be entirely consistent with a mitochondrial influx of Mg^2+ ^(see Figure 8). Binding of Mg^2+ ^to the external inhibitory site may be expected to inhibit Mg^2+ ^influx into the matrix space. However, a salt-based medium may displace inhibitory Mg^2+^. Yeast mitochondria do not swell when suspended in a 0.3 M Mg^2+ ^acetate medium [25] indicating that an influx of Mg^2+ ^into the matrix space does not occur at this very high concentration. Therefore mitochondrial influx may depend on the specific KCl and Mg^2+ ^concentrations employed. In a previous report Mg^2+ ^was transported into yeast mitochondria in the absence of a membrane potential, however at a highly reduced rate compared to energized mitochondria [30].

### Comparison to yeast mitochondrial cation transporters

Fatty acids and BSA do not regulate the mitochondrial Mg^2+ ^and Ca^2+ ^release studied in this report as they did the yeast mitochondrial fatty acid-induced Ca^2+^/H^+ ^exchanger described previously [25]. Since yeast mitochondria *in situ *are exposed to an environment containing KCl and do not appear to have a high activity Ca^2+ ^efflux pathway open [22], perhaps some regulatory element inhibiting mitochondrial Ca^2+ ^release is lost during mitochondrial isolation or upon suspension of isolated mitochondria in ionic media.

The yeast mitochondrial Mg^2+ ^release activity may be of a similar nature as the yeast mitochondrial K^+^/H^+ ^exchange. The similar inhibitors and conditions stimulating mammalian mitochondrial K^+ ^and Mg^2+ ^transport have been noted previously [12,34]. The activities of both yeast proteins are non-specific in nature [26] (and see Figure 8). Cations also stimulate both activities [26] (and see Figure 6). Further experiments are needed to determine if divalent cations can exchange for many other cations across the inner mitochondrial membrane or just for protons. Both yeast mitochondrial Mg^2+ ^release and K^+^/H^+ ^exchange appear to be inhibited by divalent cations. The yeast K^+^/H^+ ^exchange, although only moderately inhibited by Mg^2+^, is strongly inhibited by Zn^2+ ^[26,35,36], whereas the ability of chelators to activate Mg^2+ ^release suggests a role for divalent cations in regulating this activity as well. However there is at least one striking difference in the regulation of yeast mitochondrial Mg^2+ ^release and K^+^/H^+ ^exchange. The K^+^/H^+ ^exchange appears to be spontaneously active in isolated yeast mitochondria [26] when they are suspended in a mannitol medium as monitored by the efflux of endogenous K^+ ^(data not shown) while Mg^2+ ^release does not occur spontaneously in a mannitol medium.

In a previous study no loss of free mitochondrial Mg^2+ ^was detected in yeast mitochondria when they were suspended in a 0.135 M KCl medium for thirty minutes [30], while we detect a loss of over 70 percent of the total mitochondrial Mg^2+ ^levels in just ten minutes in 0.3 M KCl media using mitochondria from strain W303-1A. However, in the previous study the DBY747 yeast strain was used and the mitochondria were preloaded in a 10 mM Mg^2+ ^135 mM KCl medium before Mg^2+ ^release was monitored. It will be important to determine which of these factors is responsible for the different Mg^2+ ^efflux results.

### Yeast mitochondrial Mg^2+ ^efflux candidates

Mitochondrial matrix space Mg^2+ ^is important for many aspects of nucleotide metabolism [37,38]. Two inner mitochondrial membrane transporters, Mrs2p and Lpe10p, are needed for group II intron splicing [16,39]. *MRS2 *and *LPE10 *have slight sequence similarity with the bacterial Mg^2+^transporter CorA. Assays with a fluorescent Mg^2+ ^indicator dye indicate that Mrs2p is part of an electrophoretic mitochondrial Mg^2+ ^influx pathway inhibited by cobalt(III)hexaammine [30]. Mitochondrial Mg^2+ ^levels changed with the levels of Mrs2p and Lpe10p. Mitochondrial electrophoretic Mg^2+ ^uptake was absent in an *MRS2 *deletion strain. Mrs2p and Lpe10p are essential for yeast growth on nonfermentable carbon sources [38]. However they cannot substitute for each other suggesting non-redundant functions. It is possible that Mrs2p or Lpe10p is responsible for the mitochondrial Mg^2+ ^release described in this report. However, in the previous experiments Mg^2+ ^was taken up by energized mitochondria in an Mrs2p-dependent manner where we found that Mg^2+ ^was released from energized mitochondria [30]. In the previous study there was also no difference in the uptake of Mg^2+ ^in ionic or non-ionic media, while greatly differing Mg^2+ ^efflux results occurred in this report under these conditions. In addition cobalt(III)hexaammine, an inhibitor of Mrs2p, blocked Mg^2+ ^transport while having no effect on Ca^2+ ^transport [30], while the efflux described here appears not to discriminate between Mg^2+ ^and Ca^2+^.

The *YOL027 *and *YPR125 *genes are multicopy suppressors of the respiratory-deficient phenotype caused by deletion of *MRS2 *[30] and are therefore candidate mitochondrial Mg^2+ ^transporters. YOL027p has been demonstrated to mediate K^+^/H^+ ^exchange activity in yeast mitochondria [30]. Because of the similarities in mitochondrial Mg^2+ ^and K^+ ^transport properties mentioned above either YOL027p or YPR125p may also function as a Mg^2+^/H^+ ^exchanger. Mg^2+ ^inhibited K^+^/H^+ ^exchange activity in YOL027p-contaning submitochondrial particles. Deletion of *YOL027 *resulted in slightly altered mitochondrial transport rates of both Mg^2+ ^and Ca^2+ ^[40]. It is possible that the homologous *YPR125 *gene (40 % sequence identity) could be responsible for the remaining Mg^2+ ^and Ca^2+ ^transport activity because overexpression of *YPR125 *rescued growth of the *YOL027 *deletion strain and YPR125p also localized to mitochondria.

When added to yeast cells certain ionophores such as nigericin selectively target mitochondria [41]. Nigericin is an ionophore that possesses K^+^/H^+ ^exchange activity. A screen for nigericin-resistant mutants revealed that the *Mdm31 *and *Mdm32 *genes may be involved in mitochondrial cation homeostasis [42]. *Mdm31 *and *Mdm32 *deletion mutants contained a two to three-fold higher level of mitochondrial Mg^2+ ^than the wild type. Since yeast mitochondrial K^+^/H^+ ^exchange is inhibited by Mg^2+ ^[36,43], it was hypothesized that Mdm31p and Mdm32p may be proteins that mediate the efflux of mitochondrial Mg^2+ ^[42]. Knocking out their function could have elevated mitochondrial Mg^2+ ^levels to inhibit the endogenous mitochondrial K^+^/H^+ ^exchanger to rescue the cells from nigericin-mediated death.

### Mammalian mitochondrial Mg^2+ ^transport

We have shown that yeast mitochondria contain a Mg^2+ ^release activity, while a similar activity has been demonstrated in mammalian mitochondria [33]. Mammalian mitochondria are hypothesized to take up Mg^2+ ^through a uniport mechanism or by respiration-dependent 'unspecific leak' and release Mg^2+ ^by a Mg^2+^/H^+ ^exchange [33,44]. Our current data combined with data from the Schweyen laboratory [30] indicate that a similar system is likely present in yeast. They have characterized Mrs2 as the respiration-dependent mitochondrial Mg^2+ ^uptake system, while the efflux described in this report is likely a Mg^2+^/H^+ ^exchange. In this regard the respiratory substrate-induced release that occurs in yeast mitochondria in the absence of external Mg^2+ ^resembles the respiration-induced release in rat heart attributed to Mg^2+^/H^+ ^exchange [33]. The ionic strength-induced release of Mg^2+ ^in yeast mitochondria resembles a Mg^2+ ^release activity in rat liver mitochondria that is stimulated by alkaline pH in the presence of fatty acids [12]. This rat liver mitochondrial Mg^2+ ^release also only occurred in an ionic medium. This suggests that yeast and mammalian mitochondrial Mg^2+ ^transport may occur through similar mechanisms.

## Conclusion

By using a Ca^2+^-indicating dye and measuring mitochondrial Mg^2+ ^levels we have identified Ca^2+ ^and Mg^2+ ^efflux activities stimulated by increased ionic strength in yeast mitochondria. Mitochondrial efflux did not appear to be spontaneously active when isolated mitochondria were suspended in a sugar-containing medium. Nucleotide di- and tri- phosphates, increasing pH, and divalent cation chelators also activated mitochondrial Mg^2+ ^release. The function of this mitochondrial efflux pathway is likely important for regulating the matrix space concentrations of Mg^2+^, Ca^2+^, and perhaps other divalent cations.

## Methods

### Yeast growth and mitochondrial isolation

The yeast strain W303-1A was grown aerobically at 30°C in a media containing 2% lactate, 1% yeast extract, 2% peptone, 0.05% dextrose, and 0.01% adenine at pH 5.0. Yeast cells were harvested during logarithmic growth phase (A_600 _= 1.8–2.2). Mitochondria were isolated from spheroplasts as previously described [21,45], except 0.6 M sucrose was used in the homogenization buffer instead of 0.6 M mannitol. The isolated yeast mitochondria were suspended in 0.6 M mannitol, 20 mM HEPES (K^+^), and 0.1 mM EGTA, pH 6.8. Protein concentration was determined using a mini-Biuret method using BSA as a standard. Samples were solubilized in deoxycholate (Na^+^), which was present at a final concentration of 1% by weight.

### Spectrophotometry

For ion concentration determination atomic absorption spectrophotometry was performed using an AA-575 spectrophotometer (Varian). 2 mg of mitochondrial protein was incubated in the indicated media, spun down for 2 minutes in a microcentrifuge and the supernatant poured off. The mitochondrial pellet was solubilized with 0.6 mL 2 N perchlorate overnight. 0.5 mL was removed and diluted to 2 mL with deionized water prior to sample reading. For swelling experiments, solute permeability was monitored by light scattering using an SLM- Aminco DW-2C spectrophotometer in split beam mode at A_540 _using mitochondria suspended at 1 mg protein/ml. The uptake of Ca^2+ ^by mitochondria was followed using antipyrylazo III as an indicator of the extramitochondrial Ca^2+ ^concentration [46]. Changes in ΔA_720_-A_790 _were recorded in the DW-2C operated in the dual wavelength mode and converted to Ca^2+ ^concentration values using a calibration curve that was generated with a standard solution of Ca^2+^. Estimates of membrane potential were made using safranine O (12 μM) as an indicator. Safranine accumulation was followed spectrophotometrically, as a change in ΔA_511_-A_533_. Initial absorbances without mitochondria in the media were always set to identical values. After addition of mitochondria to the medium absorbances in different ionic strength media were shifted up or down compared to runs in the same media. However, the magnitude of the dye response was similar in the different ionic strength media. Therefore, the different runs were comparable after aligning their baseline membrane potentials after FCCP addition.

### Free ion concentration determination

Medium free ion concentrations in the presence of chelators were determined using a computer program [47].

## Authors' contributions

PB designed and performed the experiments and wrote the manuscript. DP was involved in the conception of the experiments and supervised the work.
